# The Effect of Cesarean Delivery Skin Incision Approach in Morbidly Obese Women on the Rate of Classical Hysterotomy

**DOI:** 10.1155/2013/890296

**Published:** 2013-11-20

**Authors:** Brian E. Brocato, Edwin M. Thorpe, Luis M. Gomez, Jim Y. Wan, Giancarlo Mari

**Affiliations:** ^1^Department of Obstetrics and Gynecology, University of Tennessee Health Science Center, 853 Jefferson Avenue Suite E102, Memphis, TN 38163, USA; ^2^Department of Preventive Medicine, University of Tennessee Health Science Center, 853 Jefferson Avenue Suite E102, Memphis, TN 38163, USA

## Abstract

*Objective*. To assess the risk of classical hysterotomy and surgical morbidity among women with a body mass index (BMI) greater than 40 kg/m^2^ who underwent a supraumbilical incision at the time of cesarean delivery. *Methods*. We conducted a retrospective cohort study in women having a BMI greater than 40 kg/m^2^ who underwent a cesarean delivery of a live, singleton pregnancy from 2007 to 2011 at a single tertiary care institution. Intraoperative and postoperative outcomes were compared between patients undergoing supraumbilical vertical (cohort, *n* = 45) or Pfannenstiel (controls, *n* = 90) skin incisions. *Results*. Women undergoing supraumbilical incisions had a higher risk of classical hysterotomy (OR, 24.6; 95% CI, 9.0–66.8), surgical drain placement (OR, 6.5; 95% CI, 2.6–16.2), estimated blood loss greater than 1 liter (OR, 3.4; 95% CI, 1.4–8.4), and longer operative time (97 ± 38 minutes versus 68 ± 30 minutes; *P* < .001) when compared to subjects with Pfannenstiel incisions (controls). There was no difference in the risk of wound complication between women undergoing supraumbilical or Pfannenstiel incisions (OR, 2.7; 95% CI, 0.9–8.0). *Conclusion*. In women with a BMI above 40 kg/m^2^, supraumbilical incision at the time of cesarean delivery is associated with a greater risk of classical hysterotomy and operative morbidity.

## 1. Introduction

Obesity is a common, serious health condition that poses major health risks to individuals and increases costs to society. It is estimated that 97 million adults in the United States are obese as defined as body mass index 30 or greater and approximately one-third of childbearing-age women in the United States are obese [1]. Obesity places pregnant women and their offspring at increased risk for complications such as hypertensive disorders, diabetes, cesarean delivery, macrosomia, and stillbirth [2–7]. 

As is true for any abdominal surgery, cesarean delivery presents clear challenges in obese women. In pelvic surgery, low transverse or Pfannenstiel skin incisions give adequate exposure while providing a good cosmetic result [8]. Still, the pannus may limit intraoperative exposure when using a Pfannenstiel incision; in that case a vertical incision is generally preferred. To avoid the dissection of thickened layers of adipose tissue below the umbilicus, a supraumbilical vertical incision arises as an attractive alternative approach. However, this type of incision (supraumbilical) is often associated with limited intraoperative exposure, which increases operative difficulty and surgical morbidity. There is a paucity of information regarding the risks associated with supraumbilical vertical incision at the time of cesarean. Consequently, we sought to investigate whether supraumbilical vertical cesarean incisions in women with a body mass index greater than 40 kg/m^2^ are associated with classical hysterotomy as the result of restricted intraoperative exposure and other postoperative morbidities.

## 2. Materials and Methods

Hospital records of all pregnant women with a body mass index greater than 40 kg/m^2^ who underwent cesarean delivery from January 2007 to January 2011 were reviewed after approval from The University of Tennessee Health Science Center Institutional Review Board. Both dictated and written operative reports were used to identify the types of uterine and skin incisions. Our cohort included all patients who underwent supraumbilical vertical skin incisions. Patients who underwent Pfannenstiel incisions comprised controls. The control participants were selected chronologically; cohort and controls were matched at a 1 : 2 ratio. 

Our primary outcome was the occurrence of classical hysterotomy. Assuming a prevalence of the primary outcome of 5% in the control group and considering a power of 80% and 2-sided alpha = 0.05, we estimated that a sample size of 34 subjects in each group was required for a 7-fold increase in the primary outcome among women undergoing supraumbilical vertical incisions. We also investigated the following secondary outcomes: wound complications (a composite of superficial and deep surgical site infection, wound dehiscence, or return to the operating room to address the wound), need for transfusion, postoperative length of stay more than 3 days, total minutes of surgery, drain placement, endomyometritis, and estimated blood loss of more than 1000 mL. Potential confounding variables included age, body mass index, preexisting maternal diseases, number of previous abdominal surgeries, use of surgical drains, use of preoperative antibiotics, and gestational age at delivery.

Data analysis was performed using SAS 9.3 (SAS Institute Inc., Cary, NC). Two-sample *t*-test and chi-squared or Fisher's exact test was used to compare continuous and categorical data, respectively. Multivariate logistic regression analysis was performed to account for confounding variables.

## 3. Results

During the study period, 45 women delivered infants through a supraumbilical vertical incision. Two women were excluded from the cohort group due to incomplete records. Patient demographics are listed in Table 1. Our cohort group was more likely to include older women with a higher body mass index and higher prevalence of diabetes who delivered at an earlier gestational age compared to controls. All patients received preoperative antibiotics. Placement of a surgical drain was based on the preference of the surgeon. There were no cases identified with a planned classical hysterotomy. 

Classical uterine incision was more prevalent in women who had a supraumbilical skin incision compared to controls (Pfannenstiel incision) (OR, 24.6; 95% CI, 9.0–66.8). Drain placement (OR, 6.5; 95% CI, 2.6–16.2) and estimated blood loss greater than 1000 mL (OR, 3.4; 95% CI, 1.4–8.4) occurred more frequently in women with supraumbilical incisions. Total operative time was longer among those who had a supraumbilical skin incision compared to controls (97 minutes versus 68 minutes, *P* < .001). Outcomes are listed in Table 2.

After adjusting with multivariate analysis for confounders (age, body mass index, gestational age at delivery, diabetes, tobacco use, and use of a surgical drain), supraumbilical incision remained as an independent significant risk factor for undergoing classical hysterotomy, placement of a surgical drain, estimated blood loss more than 1000 mL, and longer operative time (Table 3). 

## 4. Discussion

Women with a body mass index greater than 40 kg/m^2^ who have a supraumbilical vertical skin incision at the time of cesarean are more likely to undergo a classical hysterotomy than those who have a Pfannenstiel incision. Supraumbilical incisions are also associated with a greater risk of placement of a surgical drain, estimated blood loss more than 1000 mL, and longer operative times when compared to Pfannenstiel skin incisions. 

One of the strengths of this study is the number of patients derived from a single institution over a relatively short period of time; this may limit the possibility of different techniques and postoperative care practices. The limitations of our study are those inherent to retrospective designs. Our cohort subjects differed significantly from controls in age and prevalence of higher body mass index and diabetes; however, after adjusting for these and other confounders, our results showed that supraumbilical incision was an independent risk factor that led to classical hysterotomy incision. There is also potential of referral bias given that all the patients in this study were from one regional perinatal center. The majority of the patients was of African American ancestry and had public insurance, which could limit the external validity of the study. 

Our findings agree with those reported by other investigators. Alanis et. al. showed that women with body mass index greater than 40 kg/m^2^ with vertical skin cesarean incisions were more likely to undergo classical hysterotomy; however, they did not differentiate between supraumbilical and infraumbilical vertical incisions [9].

The morbidity associated with classical cesarean delivery and women with a prior classical hysterotomy has been widely reported [10, 11]. Obesity has been shown to be an independent risk factor for cesarean delivery [12]. As the prevalence of morbid obesity rises, obstetricians caring for these women during pregnancy will be faced with decisions regarding the best surgical approach should these patients require cesarean delivery. We believe that our results provide support for advocating Pfannenstiel skin incisions in women with body mass index greater than 40 kg/m^2^ when access to the lower abdomen is feasible. Other investigators support this approach as well despite the traditional concept that intraoperative exposure is restricted whenever using Pfannenstiel incisions [9].

## 5. Conclusions

In conclusion, supraumbilical vertical approach in women with body mass index greater than 40 kg/m^2^ undergoing cesarean section is associated with higher rate of classical hysterotomy, greater blood loss, and longer operative time when compared to Pfannenstiel incisions. The importance of these findings allows clinicians to properly counsel these women who undergo cesarean delivery.

## Figures and Tables

**Table 1:** Demographics.

Characteristics	Supraumbilical *N* = 43	Pfannenstiel *N* = 90	*P* value
Age	32 ± 5	26 ± 6	<.0001
Average body mass index	64 ± 10	56 ± 6	<.0001
Gestation at delivery	36.5 ± 2.9	38.3 ± 3	.0015
African American	37 (86%)	77 (86%)	.6655
Diabetes	21 (49%)	8 (9%)	<.0001
Hypertension	23 (53%)	39 (43%)	.2722
Preeclampsia	19 (44%)	31 (34%)	.2780
Tobacco use	4 (9%)	10 (11%)	1.0000
Previous abdominal surgery	21 (49%)	44 (49%)	.9956

**Table 2:** Outcomes.

Outcome	Supraumbilical *N* = 43	Pfannenstiel *N* = 90	OR	95% CI	*P* value
Classical hysterotomy	29 (67%)	7 (8%)	24.6	9.0–66.8	<.0001
Wound complication	8 (19%)	7 (8%)	2.7	.9–8.0	.0811
Blood transfusion	7 (16%)	5 (6%)	3.3	.98–11.1	.0557
Length of stay >3 days	33 (77%)	59 (66%)	1.7	.8–4.0	.1912
Endomyometritis	2 (5%)	2 (2%)	2.1	.3–15.8	.5945
Drain placed	18 (42%)	9 (10%)	6.5	2.6–16.2	<.0001
Estimated blood loss >1 liter	14 (33%)	11 (12%)	3.4	1.4–8.4	.0055
Total minutes of surgery	97 ± 38	68 ± 30			<.0001

**Table 3:** Multivariate regression analysis.

Outcome	OR	95% CI	*P* value
Classical hysterotomy	15.8	4.1, 60.8	<.0001
Drain	7.8	2.3, 26.7	.0010
Estimated blood loss >1 liter	6.4	2.0, 20.1	.0022
Total minutes of surgery	Slope = 27.5		.0011

Variables controlled for include age, body mass index, gestational age at delivery, and diabetes mellitus.
